# Possible Deslanoside‐Induced Left Posterior Fascicular Ventricular Tachycardia: A Case Report

**DOI:** 10.1111/anec.70166

**Published:** 2026-03-28

**Authors:** Zhang Wenjun, Chen Yao

**Affiliations:** ^1^ Department of Cardiology Fuling People's Hospital of Chongqing Chongqing China

**Keywords:** atrial fibrillation, Deslanoside, electrocardiography, left posterior fascicular ventricular tachycardia

## Abstract

Cardiac glycosides, including Deslanoside, are widely used for ventricular rate control in atrial fibrillation (AF). We report a 73‐year‐old man with paroxysmal AF and no structural heart disease who developed left posterior fascicular ventricular tachycardia (LPFVT) shortly after intravenous Deslanoside administration. A close temporal relationship was observed between drug administration and arrhythmia onset, with a shorter latency following a second intravenous dose. These findings suggest that Deslanoside may have facilitated LPFVT. This case highlights that cardiac glycosides, while effective for rate control, may occasionally precipitate Purkinje‐related ventricular tachycardia.

## Graphical abstract

A 73‐year‐old man with paroxysmal AF and no structural heart disease who developed left posterior fascicular ventricular tachycardia (LPFVT) shortly after intravenous Deslanoside administration for rate control. Highlights: cardiac glycosides, though effective for rate control, may occasionally precipitate Purkinje‐related ventricular tachycardia.

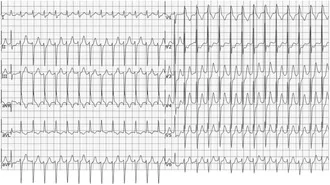

## 1 Introduction

Deslanoside is a cardiac glycoside that inhibits the Na^+^/K^+^‐ATPase pump, thereby increasing intracellular calcium concentration and enhancing myocardial contractility. Clinically, it is used primarily for the treatment of congestive heart failure and for ventricular rate control in patients with atrial fibrillation (AF) or atrial flutter. The electrophysiological effects of Deslanoside on the heart are complex, and ventricular arrhythmias, including ventricular tachycardia, are well‐recognized adverse effects of digitalis toxicity (Ershad et al. 2025). However, to our knowledge, left posterior fascicular ventricular tachycardia (LPFVT) associated with Deslanoside has rarely been reported.

## 2 Case Presentation

**FIGURE 1:**
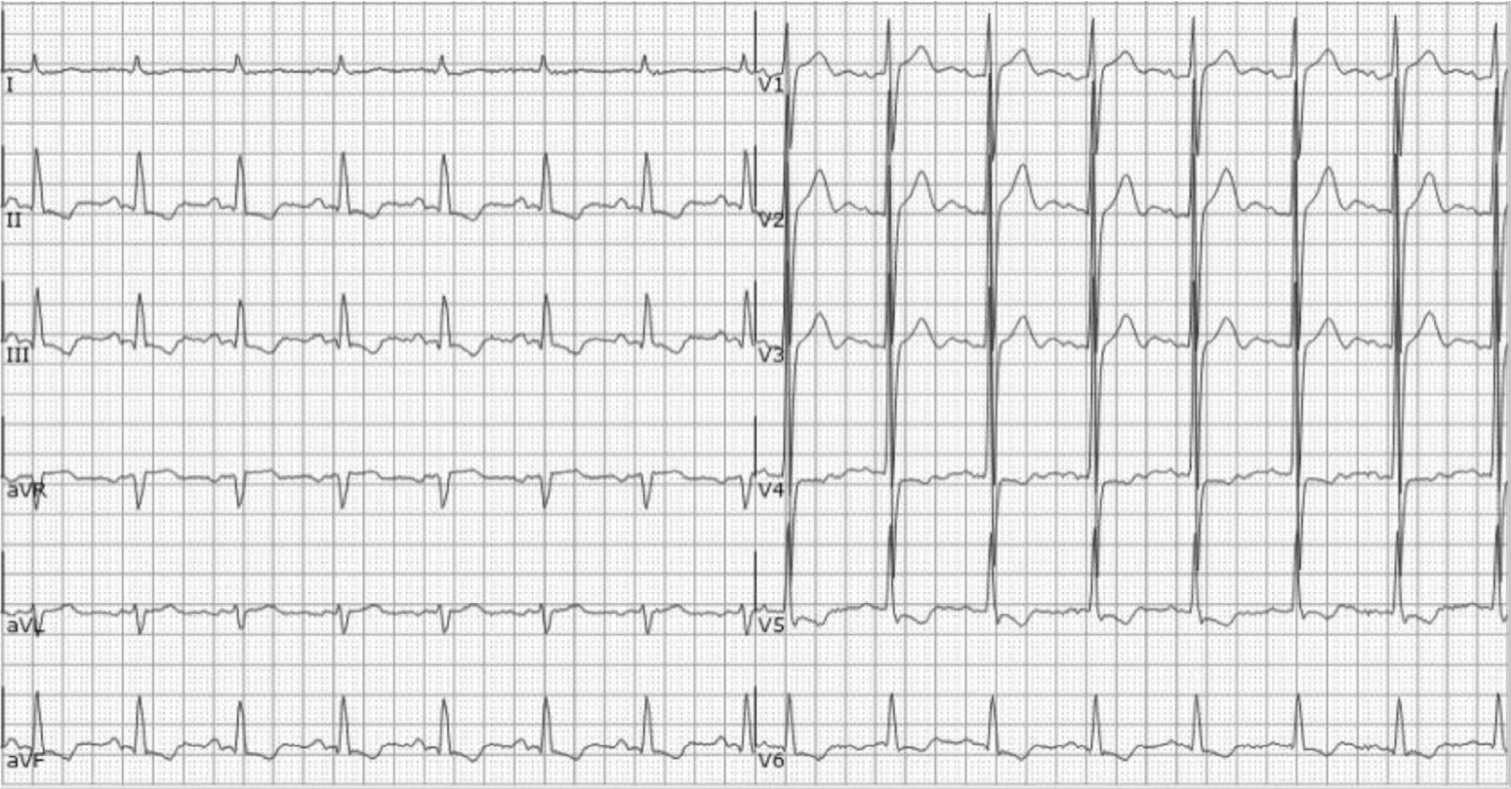
Admission 12‐lead electrocardiogram showing sinus rhythm with T wave inversion in lead II, III, aVF and V4–V6.

A 73‐year‐old man was admitted with a 2‐day history of dizziness and fatigue of lower extremities. On admission, his temperature was 38.5°C, blood pressure was 138/76 mmHg, and heart rate was 96 bpm. A 12‐lead electrocardiogram (ECG) (Figure 1) demonstrated sinus rhythm with T‐wave inversions in leads II, III, aVF and V4‐V6. Transthoracic echocardiography revealed normal cardiac structure and preserved cardiac function. Brain magnetic resonance imaging was consistent with acute cerebral infarction. Chest computed tomography showed findings suggestive of pulmonary infection. Laboratory tests revealed normal electrolyte levels, serum creatinine, and B‐type natriuretic peptide. Cardiac biomarkers, including troponin I, creatine kinase–MB, and myoglobin, were within normal limits. Given the fever and imaging findings suggestive of infection, the patient received anti‐infective and supportive therapy, along with standard treatment for acute cerebral infarction.

**FIGURE 2:**
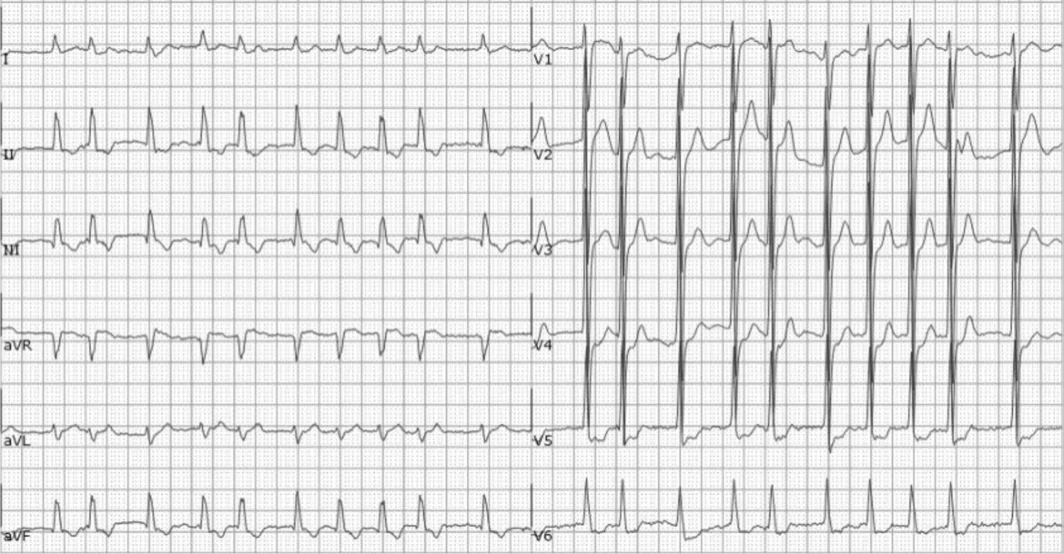
Atrial fibrillation with narrow QRS complexes. The QRS morphology is identical to that observed during sinus rhythm.

**FIGURE 3:**
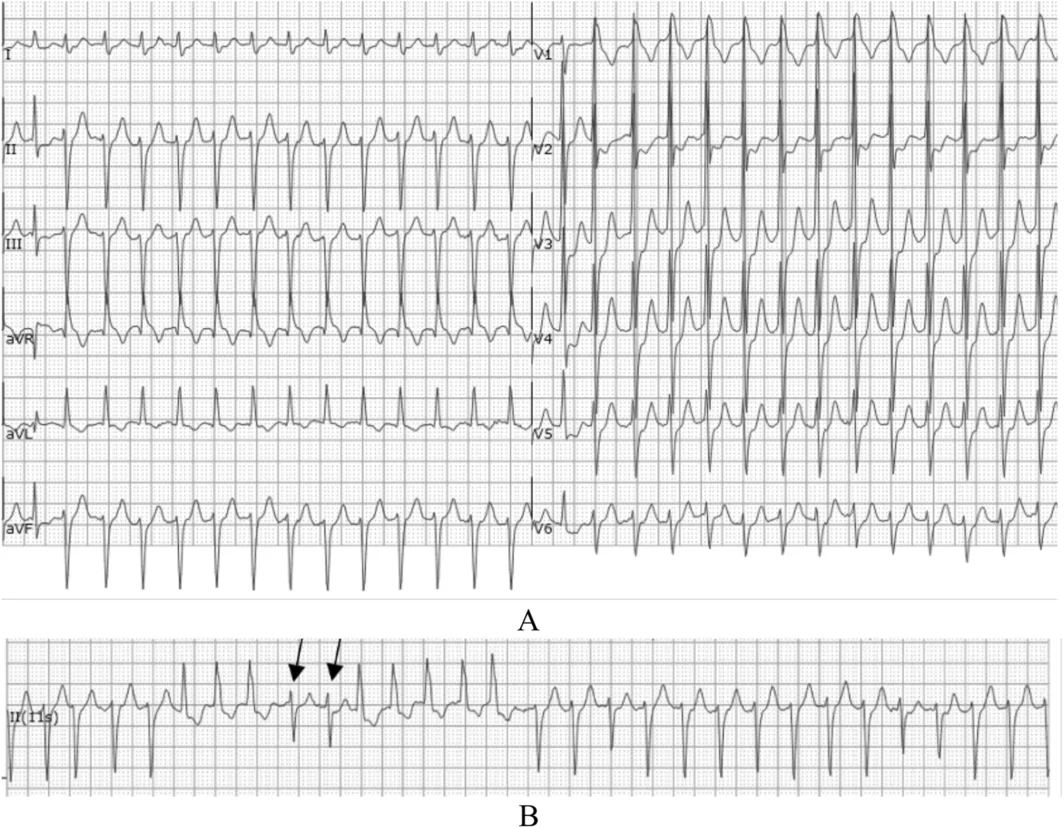
(A) Twelve‐lead electrocardiogram showing relativly wide QRS complex tachycardia without visible P waves, left‐axis deviation, and a right bundle branch block pattern in lead V1. (B) Lead II tracing showing varying QRS morphologies; black arrows indicate ventricular fusion beats.

**FIGURE 4:**
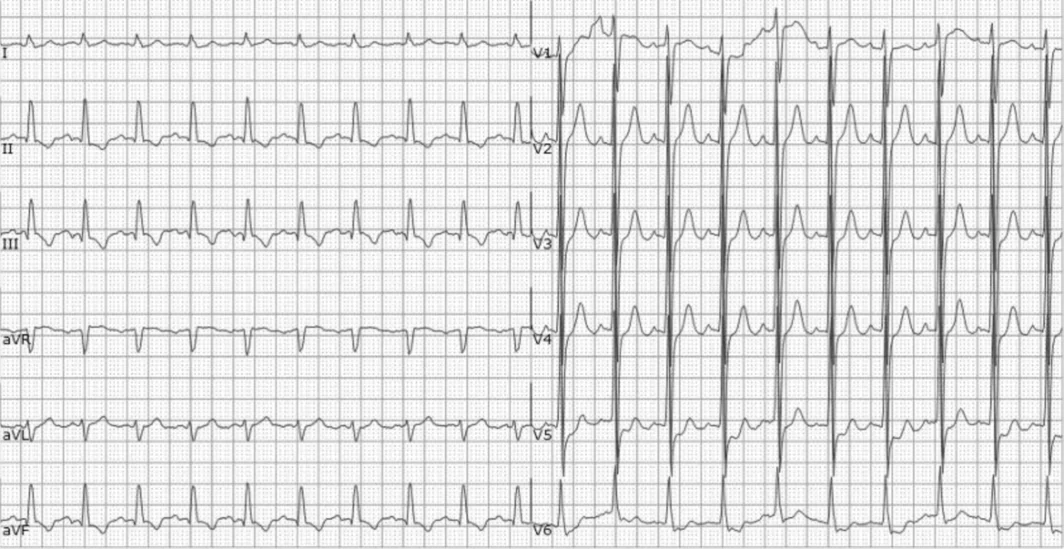
Twelve‐lead electrocardiogram obtained after cardioversion showing sinus rhythm with T wave inversion in leads II, III, and aVF. QRS morphology is identical to that in Figure 1.

Four days later, the patient developed palpitations. Electrocardiographic monitoring revealed rapid AF with a ventricular rate of 170–180 bpm. To control the ventricular rate, 0.2 mg of Deslanoside was administered intravenously. Within minutes, the palpitations were relieved, and the ventricular rate decreased to 120–130 bpm (Figure 2). Approximately 2 h later, the patient developed altered mental status, accompanied by a heart rate of 180–190 bpm, blood pressure of 64/32 mmHg, and oxygen saturation of 60%. A repeat ECG (Figure 3A) showed a relatively wide QRS tachycardia with a right bundle branch block (RBBB) pattern, left axis deviation, and occasional ventricular fusion beats (black arrows, Figure 3B). Given the presence of hemodynamic instability, immediate electrical cardioversion was performed, resulting in restoration of sinus rhythm (Figure 4). After cardioversion, the heart rate was 100 bpm and blood pressure was 110/62 mmHg, with an oxygen saturation at 82% and a respiratory rate of 27 breaths/min. Arterial blood gas analysis revealed a partial pressure of oxygen of 52 mmHg. The patient was subsequently transferred to the intensive care unit for further management.

**FIGURE 5:**
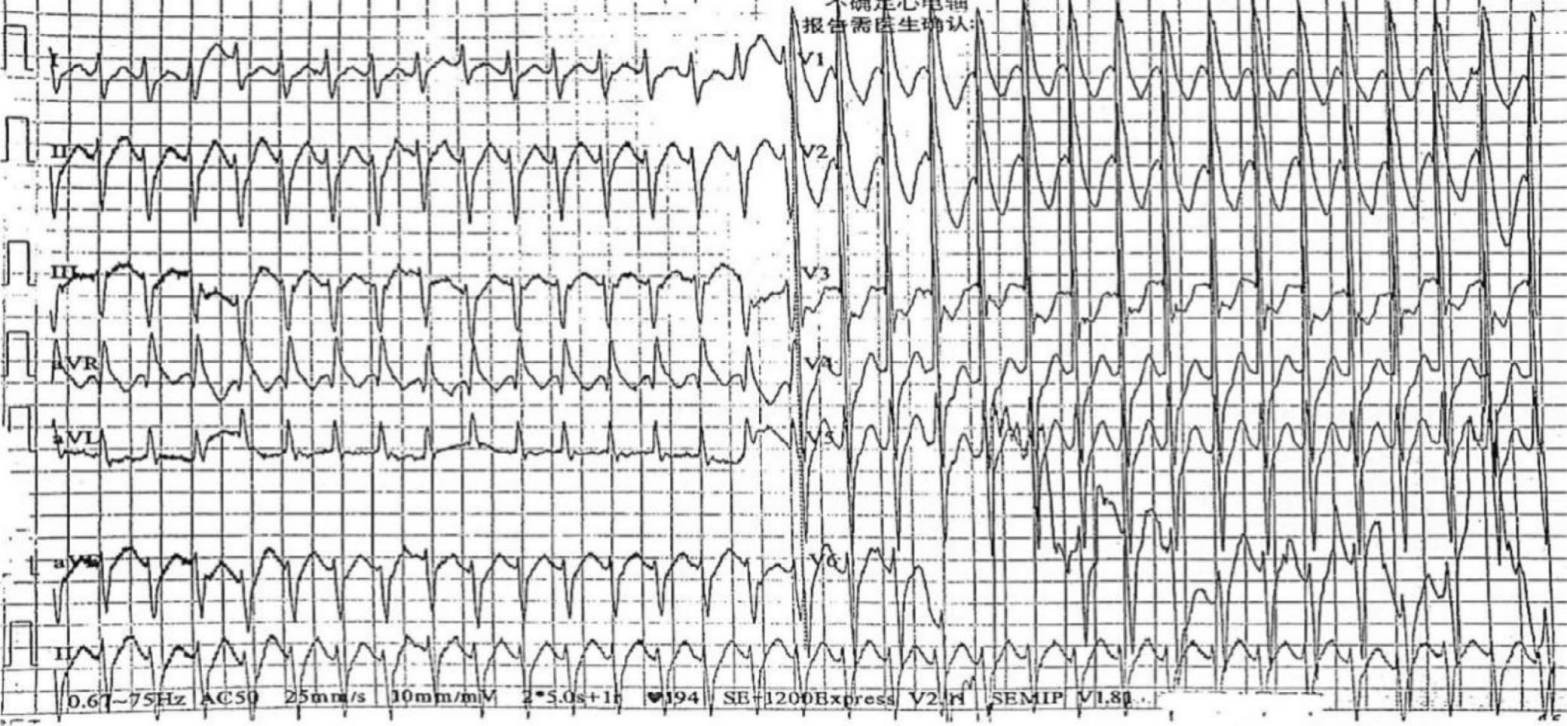
Twelve‐lead electrocardiogram showing regular, relatively wide QRS complexes with a ventricular rate of approximately 190 bpm. The QRS morphology is similar to that in Figure 3A.

**FIGURE 6:**
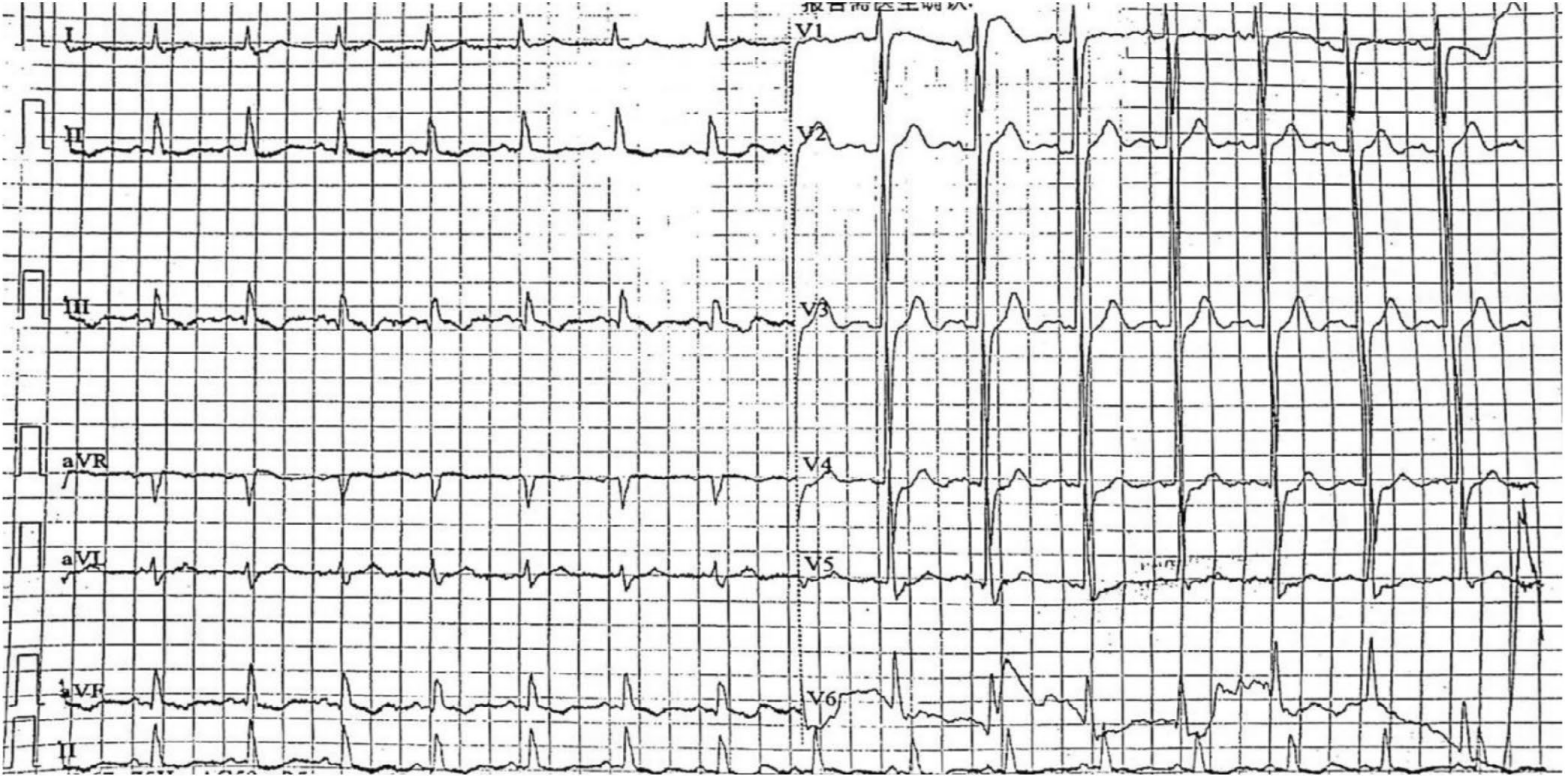
Twelve‐lead electrocardiogram showing sinus rhythm. The QRS morphology is similar to that in Figure 1.

Three days later, the patient again developed palpitations and dyspnea. An ECG initially showed AF with a ventricular rate of approximately 170 bpm. For ventricular rate control, a second intravenous dose of 0.2 mg of Deslanoside was administered. However, the ventricular rate failed to decrease and instead increased to 190 bpm within 30 min. A subsequent ECG (Figure 5) revealed a regular, relatively wide QRS tachycardia, consistent with the previously observed LPFVT pattern. Electrical cardioversion was performed, successfully restoring sinus rhythm (Figure 6). The patient's symptoms subsequently improved, and he was later transferred to another hospital at the request of his family. At the time of transfer, no additional oral antiarrhythmic or rate‐controlling medications had been initiated.

## 3 Discussion

LPFVT is a form of idiopathic left ventricular tachycardia involving the Purkinje system. Its diagnostic features include: (1) inducibility by atrial or ventricular premature stimulation; (2) an ECG pattern of right bundle branch block (RBBB) with left axis deviation; (3) absence of structural heart disease; and (4) termination by verapamil (Zipes et al. 1979; Belhassen et al. 1981).

Typical ECG characteristics include: (1) a relatively narrow QRS complex (< 120–140 ms) with RBBB morphology; (2) a leftward QRS axis, characterized by negative deflections in leads II, III, and aVF, and positive in lead aVL; and (3) occasional fusion or capture beats (Prystowsky et al. 2012; Lerman et al. 1997). In the present case, the QRS complexes were approximately 100 ms wide with a consistent RBBB pattern and left axis deviation, fulfilling the typical electrocardiographic features of LPFVT. In the early portion of Figure 3B, mild cycle length variability was observed, likely reflecting the initiation phase of the tachycardia, during which transient fluctuations in cycle length may occur before the ventricular rhythm becomes fully stabilized.

The differential diagnosis includes supraventricular tachycardia with RBBB and left anterior hemiblock (LAHB), as well as posterior papillary muscle ventricular tachycardia (PPMVT). According to the Michowitz prediction model, the probability of LPFVT in this patient was 95.8%, favoring this diagnosis over SVT with RBBB and LAHB (Michowitz et al. 2017). Moreover, the relatively narrow QRS duration (100 ms) further supports LPFVT, as PPMVT typically presents with a QRS duration greater than 160 ms.

The mechanism of LPFVT is generally considered to involve reentry within the Purkinje system. A slow conduction zone, composed of specialized Purkinje fibers in which diastolic Purkinje potentials (DPs) can be recorded, forms the antegrade limb of the reentrant circuit, whereas the left posterior fascicle and surrounding myocardium form the retrograde limb (Chen et al. 2020). Nevertheless, focal mechanisms related to triggered activity have occasionally been reported (Wieland and Marchlinski 1986).

As a cardiac glycoside, Deslanoside exerts complex electrophysiological effects. It decreases sinoatrial node automaticity, slows atrioventricular nodal conduction, and prolongs its refractory period, while shortening atrial refractory periods. Importantly, cardiac glycosides have been shown to enhance Purkinje fiber excitability, through increased automaticity and calcium‐dependent triggered activity (Rosen 1985), and may also alter Purkinje refractoriness, thereby increasing susceptibility to ventricular arrhythmias (Xie et al. 2001). Although ventricular tachyarrhythmias are recognized as manifestations of cardiac glycosides, reports linking cardiac glycosides to LPFVT remain limited, and an association with Deslanoside has not been specifically described (Wieland and Marchlinski 1986).

In the present case, both episodes of LPFVT occurred shortly after intravenous Deslanoside administration in the setting of AF. Although rapid AF has been reported to create an arrhythmogenic milieu and may serve as a trigger for idiopathic left ventricular tachycardia, it alone is unlikely to fully account for the observed arrhythmia. The close temporal relationship between Deslanoside administration and LPFVT onset, together with the shorter latency following the second intravenous dose, supports a Deslanoside‐associated facilitating effect. Based on its known effects on Purkinje fibers, we hypothesize that Deslanoside may increase the automaticity of specialized Purkinje fibers and facilitate delayed afterdepolarization–related triggered activity, thereby generating premature depolarizations that could initiate a reentrant circuit. In addition, Deslanoside may alter the refractory period of Purkinje fibers, creating electrophysiological heterogeneity that favors the establishment of reentry. In the presence of AF, such Purkinje‐related triggered activity may lower the threshold for initiating a reentrant fascicular tachycardia. Furthermore, by prolonging atrioventricular nodal refractoriness, Deslanoside may reduce interference from competing atrial impulses during AF, thereby perpetuating LPFVT. Unfortunately, electrophysiological testing could not be performed to verify this mechanism.

Notably, this presentation does not conform to the features of typical digitalis toxicity. Several observations support this distinction: (1) the patient received a single low intravenous dose of Deslanoside (0.2 mg) without prior chronic exposure to cardiac glycosides, and renal function and electrolyte levels were normal; (2) classical manifestations of digitalis toxicity such as gastrointestinal symptoms, visual disturbances, or multiple concomitant arrhythmias (e.g., bidirectional VT or atrioventricular block) were absent; (3) the observed ventricular tachycardia exhibited features of LPFVT rather than the polymorphic or bidirectional VT usually seen in digitalis poisoning; (4) the arrhythmia was promptly terminated by electrical cardioversion, consistent with Purkinje reentry VT rather than sustained digitalis‐induced arrhythmia. Following restoration of sinus rhythm, no recurrent ventricular tachycardia or ventricular tachycardia storm was observed, which may be explained by stabilization of the electrophysiological milieu such that Deslanoside exposure alone was less likely to reinitiate ventricular tachycardia. Taken together, this episode is more appropriately interpreted as Deslanoside‐ associated LPFVT rather than classic digitalis toxicity.

## 4 Conclusion

Although cardiac glycosides such as Deslanoside are effective for controlling ventricular rate in patients with rapid atrial fibrillation, their use may warrant caution, particularly in individuals with a history of LPFVT, given the potential risk of facilitating ventricular tachycardia. As this report represents a single observational case, further studies are required to elucidate the electrophysiological mechanisms underlying Deslanoside‐associated LPFVT.

## Author Contributions

Zhang Wenjun wrote the manuscript. Chen Yao reviewed the manuscript. All authors approved the final manuscript and agreed to their contributions.

## Funding

This work is supported by the Joint Medical Research Project of the Science and Technology Commission and the Health Commission of Fuling District, Chongqing, China (No. 2022KWLH073, No. 2022KWLH079).

## Conflicts of Interest

The authors declare no conflicts of interest.

## Data Availability

Research data are not shared.
